# Randomized, double-blind, two-way crossover bioequivalence and adhesion study, in healthy women, of a transdermal contraceptive patch with a newly sourced adhesive component at the end of shelf life vs. the EVRA patch at the beginning of shelf life 

**DOI:** 10.5414/CP204034

**Published:** 2021-11-15

**Authors:** Madhu Sanga, Subusola Vaughan, Julius Nangosyah, Veronika Scholz, Sergio Fonseca

**Affiliations:** 1 Janssen BioPharma, Inc., South San Francisco, CA,; 2 Janssen Research & Development, Titusville, NJ, USA,; 3 Janssen Research & Development, Beerse, Belgium, and; 4 Charité Research Organisation GmbH, Berlin, Germany

**Keywords:** bioequivalence, adhesion, contraceptive transdermal patch, pharmacokinetics, randomized controlled trial

## Abstract

Objective: Evaluate bioequivalence, based on norelgestromin (NGMN) and ethinyl estradiol (EE) plasma concentrations, and adhesion of a transdermal contraceptive patch containing a newly sourced adhesive component (test) at end of shelf life (EOSL) vs. the marketed EVRA patch (reference) at beginning of shelf life (BOSL). Materials and methods: In this randomized, double-blind, two-way crossover study, healthy women received a single, 7-day application of test and reference patches in 4 sequences: two 11-day treatment periods separated by a 21-day washout. Assessments included NGMN and EE pharmacokinetics (PK), adhesion (per European Medicines Agency (EMA) 5-point scale), irritation potential and application-site reactions, and tolerability. Patches were bioequivalent if 90% CIs of geometric mean ratios (GMRs) of test/reference for C_max_, AUC_168h_, AUC_0–tlast_, and AUC_∞_ were 80 – 125%. Patch adhesion was comparable if ratios of geometric mean cumulative adhesion percentages were ≥ 90%. Results: 68 women were randomized, and 62 completed both treatments. 55 and 59 participants in the reference and test group, respectively, had patch adhesion ≥ 80% (EMA score 0 – 1) at end of treatment. Bioequivalence was demonstrated: GMRs for pharmacokinetic (PK) parameters ranged from 102.76 to 105.57% for NGMN and 93.78 – 94.80% for EE, and associated 90% CIs were fully within the bioequivalence acceptance range (80 – 125%) for both. The patches had comparable adhesion properties (GMR, 101.4% (90% CI: 99.2 – 103.6)) and incidences of treatment-emergent adverse events. Conclusion: NGMN-EE transdermal test patch at EOSL was bioequivalent to the marketed patch at BOSL, supporting widening the product’s shelf-life specification. Adhesive properties and safety profiles were comparable between patches.

Trial registration: EudraCT Number 2019-001893-27.

**What is known about this subject**

- Previous clinical research has demonstrated that the EVRA transdermal contraceptive patch containing progestin and estrogen is efficacious and convenient. The patch contains 6 mg of norelgestromin (NGMN) and 600 μg of ethinyl estradiol (EE), with mean daily release rates of ~ 203 and ~ 33.9 mg. Steady-state concentrations (C_ss_; ~ 0.8 ng/mL and ~ 50 pg/mL) were achieved within 48 hours of a single application, and rates were maintained throughout the recommended 7-day wear period.
- The EVRA patch is comprised of 3 layers: a contact adhesive/drug-containing layer, an occlusive backing layer to cover and protect the adhesive/drug layer, and a disposable polyester film that protects the contact layer and is removed before use. The currently marketed formulation of the adhesive component, Oppanol B100, has been discontinued and is being replaced with Oppanol N100.
- A previous study in 70 healthy women showed bioequivalence for NGMN and EE in newly manufactured patches containing Oppanol N100. It was not determined if the patches would be bioequivalent at the end of their shelf life.

**What this study adds**

- This study evaluated hormone exposure, adhesion, and tolerability of the new test patch (Oppanol N100) at the end of shelf life (EOSL) compared to the currently marketed reference patch (Oppanol B100) at the beginning of shelf life (BOSL) in 68 healthy women. The results of this clinical study justify the widening of the currently approved EVRA’s drug product shelf-life specification.
- PK analysis demonstrated bioequivalence for NGMN and EE exposure between the test patch at EOSL and the reference patch at BOSL. The test patch was non-inferior to the reference patch for adhesion.
- The test and reference patches had comparable tolerability and safety profiles, with similar rates of skin reaction, irritation, and adverse events.

## Introduction 

Previous clinical research has demonstrated that a transdermal contraceptive patch containing progestin and estrogen is efficacious and convenient [1, 2]. Thus, its use is associated with improved compliance compared with oral contraceptives [3, 4, 5]. EVRA (RWJ10553) is a 20-cm^2^ transdermal contraceptive patch that is used once weekly for 3 consecutive weeks followed by a patch-free week. EVRA is approved for use in Asia, Canada, Europe, and Latin America. The patch contains 6 mg of the progestin norelgestromin (NGMN) and 600 μg of the estrogen ethinyl estradiol (EE), with mean daily release rates of ~ 203 and ~ 33.9 mg/day, respectively [6]. C_ss_ of ~ 0.8 ng/mL and ~ 50 pg/mL, respectively, were achieved by 48 hours after a single application, and rates were maintained throughout the recommended wear period of 7 days [7, 8]. 

The EVRA contraceptive patch is comprised of 3 layers: a contact adhesive/drug-containing layer, a colored occlusive backing layer to cover and protect the adhesive/drug layer, and a disposable polyester film that protects the contact layer and is removed before use. In addition to the hormonal components of the patch, the adhesive/drug layer contains several inactive components (crospovidone, polyisobutylene (PIB)/polybutene, non-woven polyester fabric, and lauryl lactate) to provide good adhesive properties and to facilitate drug delivery through the skin. The high-molecular-weight (HMW) PIB constitutes 6% of the total adhesive formula. The currently marketed formulation uses the HMW PIB Oppanol B100, which has been discontinued. Thus, there is a need to replace the Oppanol B100 with a similar component, HMW PIB Oppanol N100, from the same manufacturer. All other excipients and active components remain unchanged. 

A previous bioequivalence study [9] was conducted in 70 healthy female participants to compare exposures of NGMN and EE in the new patch containing Oppanol N100 with the currently marketed patch containing Oppanol B100. Non-inferiority in adhesion of the new patch compared to the currently marketed patch was also evaluated. The results showed that the patches were bioequivalent, and non-inferiority in adhesion was confirmed [9]. The irritation potential, safety, and tolerability of the test and the reference patches were comparable. The present study was designed to evaluate hormone exposure and the adhesion of the new patch at the end of shelf life (EOSL) compared to the currently marketed patch at the beginning of shelf life (BOSL) to justify the widening of the drug product’s shelf-life specification. 

## Materials and methods 

### Objectives 

The primary objectives of the study were: (1) to determine bioequivalence of the transdermal contraceptive patch containing the newly sourced adhesive component Oppanol N100 at EOSL (~ 24 months after manufacture) vs. the currently marketed EVRA patch with the adhesive component Oppanol B100 at BOSL (~ 6 months after manufacture), based on NGMN and EE concentrations in plasma; and (2) to evaluate the adhesion of the new patch with Oppanol N100 at EOSL vs. the current EVRA patch with Oppanol B100 at BOSL. Secondary objectives were to evaluate the safety, tolerability, and irritation potential of the transdermal contraceptive patch containing Oppanol N100 at EOSL vs. the currently marketed EVRA patch with Oppanol B100 at BOSL. 

### Study participants 

Healthy women of childbearing potential aged 18 – 45 years, with body mass index (BMI) ≥ 18 and ≤ 30 kg/m^2^, and body weight ≥ 50 kg and ≤ 100 kg, were enrolled. Participants were eligible if their systolic blood pressure was between 90 and 140 mmHg and diastolic blood pressure was no higher than 90 mmHg, they had hematocrit ≥ 36%, and a 12-lead electrocardiogram consistent with normal cardiac conduction and function at screening (i.e., normal sinus rhythm with heart rate ≥ 45 and ≤ 100 bpm, QT interval corrected for heart rate according to Fridericia’s formula ≤ 470 ms, QRS interval ≤ 120 ms, and PR interval ≤ 220 ms). Participants were required to have a negative serum (β-human chorionic gonadotropin) pregnancy test at screening and a negative urine pregnancy test on day −1 of each treatment period. Participants were required to be surgically sterile with intact ovaries, sexually abstinent, or using highly efficient non-hormonal contraceptive method before admission and until 1 month after study completion. Participants were required to be non-smokers or ex-smokers for > 6 months, not use nicotine-containing substances, and test negative for cotinine at screening and on day −1 of each treatment period. Participants also were screened for the presence of human immunodeficiency virus, hepatitis B virus surface antigen, hepatitis B core antibodies, and hepatitis C virus antibody. 

### Study design 

This randomized, double-blind, multi-center, two-way crossover, phase 1 study was designed to evaluate bioequivalence and adhesion of a single, 7-day application of 1 test patch (Oppanol N100 at EOSL) and 1 reference patch (Oppanol B100 at BOSL). The study was conducted at three clinical sites (one each in Belgium, Germany, and the Netherlands) from August 7, 2019, to December 13, 2019. The study consisted of a screening phase (within 28 days of patch application on day 1 of treatment period 1), a double-blind treatment phase consisting of two single-application treatment periods separated by a 21-day washout period (starting from the day of patch removal (day 8 of treatment period 1)), and an end-of-study phase after completion of the 240-hour pharmacokinetic (PK) sampling period on day 11 of treatment period 2, or upon early withdrawal. The overall duration of study participation was ~ 67 days. 

**Figure 1:**
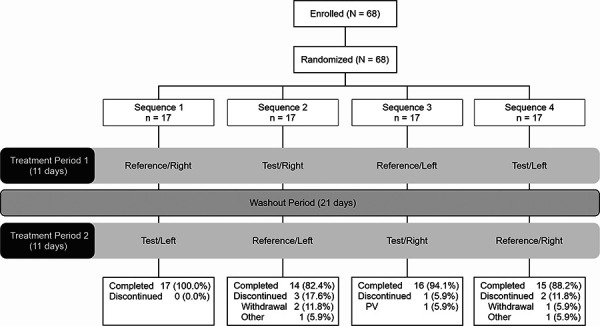
Study design and disposition. Enrolled participants were randomly assigned to 1 of 4 treatment sequences. On day 1 of each treatment period, participants received one patch (test or reference) applied to either the right or left buttock. PV = protocol violation.

A modified two-way crossover design was used to confirm that there was no application-site-related biases. Participants were randomly assigned to 1 of 4 treatment sequences based on a computer-generated randomization code using randomly permuted blocks. The 4 treatment sequences were: (1) reference/right → test/left, (2) test/right → reference/left, (3) reference/left → test/right, and (4) test/left → reference/right (Figure 1). 

### Treatment administration and assessments

Participants visited the study site on day −1 in each treatment period (≥ 10 hours before patch application) and remained at the site until collection of the 240-hour PK sample on day 11 (treatment period 1) or until completion of the end-of-study assessments after collection of the 240-hour PK samples on day 11 (treatment period 2), or upon early withdrawal. In the morning of day 1 of each treatment period, 1 patch (test or reference) was applied to the left or right buttock of each participant by a designated study-site personnel member. Residue formation was assessed on the release liner after it was removed from the patch (before patch application). 

Blood samples were collected before patch application (pre-dose) and after patch application (days 1 (0 hours), 2 (24 hours), 3 (48 hours), 4 (72 hours), 5 (96 hours), 6 (120 hours), 7 (144 hours), 8 (168 hours, 168.5 hours, 171 hours, 174 hours, and 180 hours), 9 (192 hours), 10 (216 hours), and 11 (240 hours)) for measurement of plasma concentrations of NGMN and EE, which were determined using a validated, specific, and sensitive high-performance liquid chromatography method with tandem mass spectrometric detection (LC-MS/MS) [10]. 

### Bioanalytical methodology 

As part of LC-MS/MS method validation, intra-run accuracy and precision were investigated at 7 different quality control (QC) concentration levels (25.0 and 5.00 pg/mL (lower limit of quantitation (LLOQ), 75.0 and 15.0 pg/mL, 250 and 50 pg/mL, 500 and 100 pg/mL, 1,250 and 250 pg/mL, 1,875 and 375 pg/mL, and 37,500 and 7,500 pg/mL for NGMN and EE, respectively). For calculation of accuracy and precision, the following formulas were used.


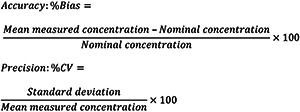


### Pharmacokinetic analyses 

All PK parameters were calculated using actual blood sampling and patch application times using non-compartmental analysis. Non-compartmental PK analysis was conducted using Phoenix WinNonlin version 8.1 (Certara L.P., Princeton, NJ, USA). PK samples with actual sampling time deviating by 5 minutes or more from the scheduled time were excluded from the calculation of descriptive statistics for plasma concentrations but included in the estimation of PK parameters. When more than half (> 50%) of the individual concentration values for a given timepoint were below the LLOQ, the mean, minimum, and median were reported as below quantification limit, while standard deviation (SD), coefficient of variation (%CV), and geometric mean were not reported. For graphical analysis, plasma concentration values below LLOQ were treated as being 0 for the plots. 

### Bioequivalence assessment 

All participants who completed both treatment periods, regardless of adhesion score, were included for assessment of bioequivalence. The key bioequivalence criteria were based on maximum observed plasma concentration (C_max_), area under the curve from time 0 to a specified timepoint post-dose (AUC_168h_ (or AUC_0–tau_)), AUC from time 0 (patch application) up to the last measurable concentration (AUC_0–tlast_), and AUC from time 0 to infinite time (AUC_∞_). Additional parameters were time to reach maximum plasma concentrations (t_max_), C_ss_ (calculated as mean concentration between 48 and 168 hours), AUC from time 0 to a specified timepoint post-dose (AUC_240h_), and apparent terminal elimination half-life (T_1/2_). These parameters have been used previously for bioequivalence assessments of the EVRA patch [9]. 

### Adhesion assessments 

Adhesion assessments were performed within 5 minutes after patch application on day 1 (baseline) and every 24 hours (± 20 minutes) after patch application up to patch removal at 168 hours (day 8). Residue formation on the patch was assessed after it was removed on day 8. Patch adhesion was assessed using the European Medicines Agency (EMA) 0 – 5 scoring system [11], where 5 indicates 0% to ≤ 50%, 4 indicates > 50 – 60%, 3 indicates > 60 – 70%, 2 indicates > 70 – 80%, 1 indicates > 80 – 90%, and 0 indicates > 90 – 100% adhesion. To document adhesion, 3 digital photographs of the skin (1 top view and 2 lateral views) were taken within 10 minutes of patch application on day 1 (baseline) and every 24 hours after patch application until patch removal on day 8. A qualitative evaluation of cold flow, such as the formation of a dark ring around the transdermal patch during use, patch movement or displacement, and wrinkling, also was performed. The skin site of patch application was monitored for reactions using a 3-point scale for the extent of erythema, edema, and pustules/papules and a 4-point scale for the severity of itching and erythema at screening, within 10 minutes before patch application, and at 30 minutes and 24 hours after patch removal. Photographs for skin irritation were taken within 30 minutes before patch application on day 1 (baseline) and at 30 minutes and 24 hours after patch removal. 

Treatment-emergent adverse events (TEAEs) were monitored throughout the study. End-of-study assessments were performed after collection of the 240-hour PK sample on day 11 of treatment period 2, or upon early withdrawal. 

### Ethics 

The study protocol and amendment were reviewed and approved by an independent Ethics Committee (Institutional Review Board, University Hospital Antwerp), and the study was conducted in accordance with the Declaration of Helsinki, Good Clinical Practice guidelines, and other applicable regulatory requirements. All participants provided written informed consent before enrollment. 

### Statistical analyses 

Based on an estimated intra-individual CV < 20% for AUC_168h_ and C_ss_ of NGMN and EE from a previous study [9], a sample size of 40 participants was sufficient to conclude bioequivalence at a 5% level of significance, with an overall power of > 95%, assuming that the test and reference treatment means differed by 5%. To achieve an overall power > 95%, a power of 98.4% of concluding bioequivalence was used for the comparison (test vs. reference) and for each of the two analytes. 

Based on the previous study [9], the estimated intra-individual %CV for cumulative adhesion percentages for EVRA patches was 25.7%. With an intra-individual CV of 25% and a sample size of 56 participants, the probability that the lower limit of the two-sided 90% confidence interval (CI) (equivalent to a one-sided 95% CI) for the ratio of the geometric mean cumulative adhesion percentages between test product and reference product was ≥ 90%, was estimated to be > 70% when the test and reference patches’ cumulative adhesion percentages means were equal. Approximately 68 healthy adult women had to be enrolled to ensure that ≥ 56 participants completed both assigned treatments. 

The PK statistical analysis set included all participants who completed both treatment periods. Statistical analysis was performed using Microsoft Excel (Microsoft, Redmond, WA, USA) and SAS (SAS Institute Inc., Cary, NC, USA). The least squares means (LSMs) and intra-participant variance were estimated using a mixed-effect analysis of variance. The ratios of geometric LSMs PK parameters and the 90% CIs were calculated for the test and reference patches; the patches were considered bioequivalent if the 90% CIs of the ratios for C_max_, AUC_168h_, AUC_0–tlast_, and AUC_∞_ were 80 – 125% [12]. 

The adhesion analysis included all randomized participants with ≥ 1 adhesion assessment. Cumulative adhesion percentages from the time of patch application to the time of patch removal was calculated for the test and reference patches. A mixed-effects model that included treatment, treatment period, treatment sequence, and application site (left or right) as fixed effects and participant as a random effect, was used to estimate the LSMs and intra-participant variance. The ratios of geometric mean cumulative adhesion values and 90% CIs were calculated, and the test patch was considered non-inferior to the reference patch if the lower limit of the 90% CI was ≥ 90% [13]. 

The number and percentage of participants with specific application-site reactions were summarized descriptively for each treatment. The safety analysis set included all randomly assigned participants who received ≥ 1 patch application and had an adhesion percentage ≥ 0 at baseline. TEAEs were summarized using descriptive statistics. 

## Results 

### Participant disposition and baseline demographics 

Overall, 68 women were enrolled and randomized to 1 of the 4 treatment sequences. Of 68 participants, 62 (91.2%) completed both treatment periods, and 6 (8.8%) discontinued prematurely (Figure 1). 

Overall, mean (SD) age of participants was 32.3 (6.8) years. Nearly 90% of participants were white, and 9% were African American. 3% were of Hispanic or Latino ethnicity. Other demographic and clinical characteristics are summarized in Table 1. Immunological laboratory analyses for human immunodeficiency virus, hepatitis B virus surface antigen, and hepatitis C virus antibody were negative for all participants. 

### Bioanalytical results

An LLOQ selectivity experiment was conducted successfully where accuracy was within ± 20.0% for at least 5 of the 6 samples, and precision was ≥ 20.0% for all samples. Intra-run accuracy and precision met the acceptance criteria of accuracy within ± 15.0% (within ± 20.0% for LLOQ) and %CV no more than 15.0% (20.0% for LLOQ). All calibration curves had a coefficient of determination (R^2^) of ≥ 0.9922 for NGMN and ≥ 0.9949 for EE. In an incurred sample re-analysis evaluation of 157 samples for NGMN and 173 samples for EE, the incurred samples met the acceptance criteria of at least 2 of 3 of all analyzed samples having no more than a ± 20.0% difference when compared to the original analysis results with 154/157 samples (98.1%) and 162/173 samples (93.6%) within acceptance for NGMN and EE, respectively. NGMN and EE were stable for at least 8 freeze/thaw cycles. The mean of the obtained concentrations at each QC concentration level was within ± 15.0% of the nominal concentrations, and the %CV was no more than 15.0%. 

## Pharmacokinetics 

A total of 68 participants had ≥ 1 patch application and ≥ 1 PK sample. In total, 67 participants comprised the test group (except for AUC_240h_ (66 participants) and AUC_0–tlast_ (66 participants) of NGMN and AUC_240h_ (66 participants), AUC_0–tlast_ (66 participants), and AUC_∞_ (63 participants) of EE), and 64 participants comprised the reference group (except for AUC_240h_ (63 participants), AUC_0–tlast_ (63 participants), and AUC_∞_ (63 participants) of NGMN and AUC_240h_ (62 participants), AUC_0–tlast _(62 participants), and AUC_∞_ (58 participants) of EE) at the end of the treatment period and had PK profiles that allowed accurate calculation of PK parameters (C_max_, C_ss_, AUC_168h_, AUC_240h_, AUC_0–tlast_, and AUC_∞_) for both NGMN and EE. Of the 68 participants, 64 completed both treatment periods and had PK profiles that allowed for accurate calculation of the PK parameters that were used in statistical analysis for bioequivalence (C_max_, C_ss_, AUC_168h_, AUC_0–tlast_, and AUC_∞_) for both NGMN and EE (except for AUC_0–tlast_ (62 participants) and AUC_∞_ (63 participants) of NGMN, and AUC_0–tlast_ (62 participants) and AUC_∞_ (55 participants) of EE). Reasons for data exclusion from PK analysis included dropouts (test: n = 1; reference: n = 4), adjusted correlation coefficient (R^2^adj) < 0.9 (reference: n = 5; test: n = 4), and %AUC extrapolation > 20% (test: n = 1; reference: n = 1). For calculation of the individual PK parameters, plasma concentrations below the LLOQ were treated as being 0 in case of occurrence before the first or after the last measurable plasma concentration. 

**Figure 2:**
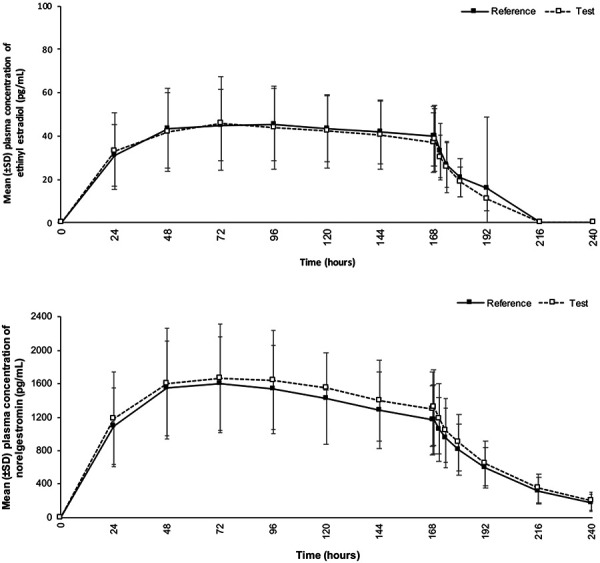
Concentration-time profiles of ethinyl estradiol (top) and norelgestromin (bottom) after a single, 7-day application of reference and test patches. Note: At 192 hours, 1 participant in the reference patch group had an outlier value of 270 pg/mL. Reference = single application of the currently marketed EVRA patch using the adhesive component Oppanol B100 at beginning of shelf life; SD = standard deviation; test = single application of the transdermal contraceptive patch using the newly sourced adhesive component Oppanol N100 at end of shelf life.

Mean plasma concentration-time profiles of NGMN and EE after a single 7-day application of the test patch were comparable to the reference patch (Figure 2). For NGMN, peak plasma concentrations occurred at 72 hours, and half-lives were comparable for the test and reference patches (mean: 27.2 and 26.8 hours; median: 26.7 and 26.6 hours) (Table 2). For EE, peak plasma concentrations (median t_max_) occurred at 96 hours, and half-lives were comparable for the test and reference patches (mean: 15.7 and 16.1 hours; median: 14.5 and 15.4 hours) (Table 2). Geometric mean ratios (GMRs) of test vs. reference patch for C_max_, C_ss_, AUC_168h_, AUC_0–tlast_, and AUC_∞_ ranged from 102.76 to 105.57% for NGMN and 93.78 to 94.80% for EE, and associated 90% CIs for the evaluated PK parameters were fully included in the bioequivalence acceptance range (80 – 125%) for both NGMN and EE (Table 3). Sensitivity analyses were also performed for participants who had evaluable PK parameters both for EE and NGMN with ≥ 1 treatment period, and statistical results were similar (bioequivalence established) to the main analysis (data on file). 

## Adhesion analysis and skin reactions 

**Figure 3:**
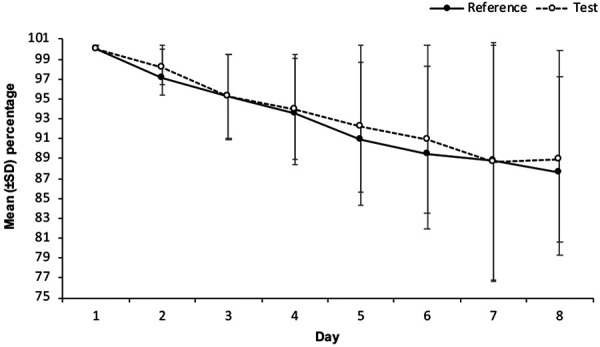
Mean estimated adhesion assessment percentages of reference and test patches (adhesion analysis set). SD = standard deviation.

Mean and median cumulative adhesion percentages were comparable between the sites of patch application (left buttock, right buttock) within each treatment group; therefore, pooled data from the two application sites were used in the analysis. Geometric mean cumulative adhesion percentage was similar for the test and the reference patches (744.3% and 734.2%, respectively). The ratio of geometric mean cumulative adhesion percentages (test/reference) was 101.4% (90% CI: 99.2 – 103.6). Because the lower limit of the 90% CI was > 90%, the test patch was deemed non-inferior to the reference patch. Adhesion percentages declined gradually over time for both test and reference patches (Figure 3). Overall, 62 participants completed both treatments with ≥ 80% adhesion (EMA score 0 – 1); ~ 50% of participants exhibited 90 – 100% patch adhesion until patch removal on day 8. One participant in the test patch group and 2 participants in the reference patch group had adhesion values ≤ 50%. 

From day 2 onward, the incidence of appearance of a dark ring around the transdermal patch, movement or displacement of the patch, and wrinkling of the patch was similar between the two treatment groups. Most (> 80%) participants in each group showed a dark ring around the patch (range: test, 86.8 – 98.5%; reference, 83.3 – 98.5%), and ~ 50% of participants in each group showed patch movement or displacement (range: test, 30.9 – 70.6%; reference, 42.4 – 72.7%). Appearance of a dark ring around the patch and movement or displacement of the patch was highest at days 5 and 6, respectively, in each group. Patch wrinkling was observed for > 50% of participants in each group, increasing over time from 44.1% on day 2 to 66.2% on day 8 in the test group and from 47.0% on day 2 to 69.7% on day 8 in the reference group. 

Overall, the test and reference patches had comparable irritation profiles. On day 8, 47% and 43% of participants in the reference- and test-patch groups had erythema (in < 50% of the occluded area). On day 9, the corresponding values were 29% and 21%, respectively. On day 8, 35% and 47% of participants in the reference- and test-patch groups, respectively, had pustules/papules (in < 50% of the occluded area). On day 9, the corresponding values were 43% and 25%, respectively. No participants had visible edema in either treatment group on days 8 and 9. Overall, the test and reference patches had comparable pruritis profiles. On day 8, 12% and 3% of participants in both treatment groups had mild and moderate itching. On day 9, 3% of participants in both treatment groups had mild itching; 2% and 0% of participants in the reference- and test-patch groups, respectively, had moderate itching. 

Residue formation on the release liner (before patch application) was not observed for either the test or the reference patches. Residue formation upon the removal of the patch at the end of the treatment periods revealed that both the test and the reference patches showed similar extent of residue formation (19 out the 67 participants had residue formation upon the removal of the patch at the end of the treatment periods). 

## Tolerability 

Overall, 91.2% of all participants reported ≥ 1 TEAE during treatment. Most TEAEs were mild in severity. In both treatment groups, TEAEs rated as moderate in severity included headache (9%), dysmenorrhea (4%), abdominal pain (3%), constipation (2%), cystitis (2%), oropharyngeal pain (2%), and presyncope (2%). No TEAEs were rated as severe. There were no discontinuations due to a TEAE during the study. TEAEs occurring in ≥ 5% of participants in both treatment groups are summarized in Table 4. TEAEs reported in > 10% of all participants were headache, breast pain, nausea, application-site erythema, abdominal pain, and fatigue. 

## Discussion 

The currently marketed EVRA patch contains Oppanol B100 as its adhesive component, which has been discontinued by the manufacturer. Oppanol N100 is the new, chemically identical component and is the only change being made to the patch. All other excipients and active components remain unchanged. In a previous bioequivalence study [9], Oppanol N100 was shown to be bioequivalent in terms of NGMN and EE exposure and non-inferior in terms of adhesion to Oppanol B100 in 70 healthy female participants using recently manufactured patches. 

This randomized, double-blind, two-way crossover study was designed to evaluate NGMN and EE exposure and adhesion of the new patch at the EOSL (~ 27 months after production) compared to the currently marketed patch at the BOSL (~ 6 months after production). For NGMN and EE, mean plasma concentration-time profiles and primary PK parameters, as measured by C_max_ and AUCs (168 h, 0–tlast, and ∞) after a single 7-day application of the test patch were similar to that of the reference patch. The 90% CIs of the GMRs for C_max_ and AUCs (168 h, 0–tlast, and ∞) were within the bioequivalence range of 80 – 125%. The intra-participant CVs were low for NGMN (11.1 – 13.4%) and EE (12.9 – 19.8%). Lower CVs yielded smaller 90% CIs for the GMRs. Plasma concentrations of NGMN and EE achieved near maximum levels within 72 and 96 hours of patch application and plateaued until patch removal at 168 hours. These t_max_ values were within the range of values reported in the previous bioequivalence study [9] and other reports [7, 8]. 

Overall, the test and reference patches showed comparable adhesion properties as demonstrated by the similar cumulative adhesion percentages. GMR for cumulative adhesion percentage (test : reference) was selected as the method of adhesion assessment, as this method is recommended by regulatory authorities. The point-estimate of the ratio of geometric mean cumulative adhesion percentages and lower limit of 90% CI for the ratios of the geometric mean cumulative adhesion percentages demonstrated similarity of adhesion between test and reference patches. For both the test and reference patches, ~ 50% of participants exhibited 90 – 100% adhesion until patch removal on day 8. In the previous bioequivalence study, a larger percentage (~ 80%) of participants exhibited 90 – 100% adhesion until patch removal [9]. The discrepancy in the proportion of participants who had 90 – 100% patch adhesion between the two studies was investigated. For both studies, various parameters were evaluated, including peel force and in vitro adhesive strength. The purpose of the peel force test was to assure that the release liner can be easily removed prior to application. The adhesive strength is defined as the force required for peeling off a patch from a stainless-steel plate under standardized conditions. All batches used in the two clinical studies gave similar results for peel force and in vitro adhesive strength within the expected analytical variability, and the results were well centered between the lower and upper acceptance criteria (data on file). Hence, the cause for this discrepancy in the proportion of participants who had 90 – 100% patch adhesion is not clear, but we assumed that – even though the evaluation of adhesion was performed by trained study personnel and according to the EMA scoring system – it is plausible that the assessments themselves could have been subject to personnel bias. In both the present and previous study [9], adhesion percentages decreased over time. 

Residue formation on the release liner (before patch application) was not observed for either the test or the reference patches. Residue formation upon the removal of the patch at the end of the treatment periods revealed that both the test and the reference patches showed similar extent of residue. 

Overall, the test and reference patches showed similar irritation profiles. The extent of erythema and pustules/papules were similar in the test- and reference-patch groups, and no edema was visible for most participants in either group. Thus, the irritation potentials were low for both the test and reference patches. 

Most participants in both the test- and reference-patch groups experienced ≥ 1 TEAE, which were mostly mild or moderate in severity. The TEAEs reported for > 10% of participants in both treatment groups were headache, breast pain, nausea, application site erythema, abdominal pain, and fatigue. There were no discontinuations owing to a TEAE, serious TEAEs, or deaths reported during the study. Overall, the test and reference patches had comparable safety profiles. 

## Conclusion 

The test patch containing the new adhesive component, Oppanol N100, at EOSL was bioequivalent to the reference patch containing Oppanol B100 at BOSL for plasma NGMN and EE concentrations, supporting the widening of the drug product’s shelf-life specification. The test patch containing Oppanol N100 at EOSL was non-inferior to the reference patch containing Oppanol B100 at BOSL for adhesion. Both patches had a low irritation potential. A single 7-day application of the test patch containing Oppanol N100 at EOSL was safe and well tolerated; neither the test nor reference patch elicited any clinically relevant safety signals. 

## Acknowledgment 

We thank all the women who participated in this study. We also thank Willi Simmler for his contributions to the study. Writing assistance was provided by Linda Goldstein and Maribeth Bogush, PhD funded by Janssen Global Services, LLC. 

## Authors’ contributions 

Madhu Sanga contributed to data collection, data analysis, data interpretation, and writing of the manuscript. Subusola Vaughan was the study medical monitor/study responsible physician and contributed to study design, data analysis, data interpretation, and writing of the manuscript. Julius Nangosyah contributed to study design, data analysis, and writing of the manuscript. Veronika Scholz contributed to data collection, data interpretation, and writing of the manuscript. Sergio Fonseca contributed to study design, data interpretation, writing of the manuscript, and portfolio study strategy. All authors met ICMJE criteria for authorship. 

## Funding 

This study was funded by Janssen R&D, LLC. 

## Conflict of interest 

M. Sanga, S. Vaughan, J. Nangosyah, and S. Fonseca report employment with Janssen R&D and stock ownership in Johnson & Johnson. V. Scholz has no conflict of interest to disclose. 

**Table 1.** Baseline demographics (safety set*).

	Sequence^†^				
Characteristic	1 (n = 17)	2 (n = 17)	3 (n = 17)	4 (n = 17)	Total (N = 68)
Age, mean (SD), y	29.9 (6.1)	31.4 (7.5)	34.1 (7.0)	33.6 (6.4)	32.3 (6.8)
Race, n (%)					
White	16 (94.1)	16 (94.1)	14 (82.4)	15 (88.2)	61 (89.7)
Black/African American	1 (5.9)	1 (5.9)	2 (11.8)	2 (11.8)	6 (8.8)
Multiple	0	0	1 (5.9)	0	1 (1.5)
Ethnicity, n (%)					
Hispanic/Latino	0	0	2 (11.8)	0	2 (2.9)
BMI, mean (SD), kg/m^2^	22.8 (2.7)	23.5 (2.9)	23.7 (2.7)	24.1 (3.2)	23.5 (2.8)
Tobacco/nicotine use, n (%)					
Former	5 (29.4)	5 (29.4)	6 (35.3)	5 (29.4)	21 (30.9)
Never	12 (70.6)	12 (70.6)	11 (64.7)	12 (70.6)	47 (69.1)

*All randomly assigned participants who received ≥ 1 patch application and had adhesion ≥ 0% at baseline. ^†^Sequence 1: reference right/test left; sequence 2: reference right/test left; sequence 3: reference left/test right; sequence 4: reference left/test right. BMI = body mass index; reference = single application of the currently marketed EVRA patch using Oppanol B100 at beginning of shelf life; SD = standard deviation; test = single application of the transdermal contraceptive patch using Oppanol N100 at end of shelf life.

**Table 2.** Plasma PK parameters of ethinyl estradiol and norelgestromin after single, 7-day application of EVRA (PK analysis set*).

	Ethinyl estradiol		Norelgestromin	
	Reference patch^†^	Test patch^‡^	Reference patch^†^	Test patch^‡^
Participants	64	67	64	67
C_max_ (pg/mL)				
Mean (SD)	52.3 (32.2)	49.7 (21.7)	1,659 (549)	1,755 (641)
Median (min, max)	47.4 (18.9, 270)	49.1 (18.5, 127)	1,610 (722, 3,380)	1,730 (713, 3,680)
Geometric mean	47.4	45.6	1573	1,642
t_max_ (h)				
Median (min, max)	96.0 (24.0, 192.0)	96.0 (24.0, 168.5)	72.0 (48.0, 168.5)	72.0 (48.0, 168.5)
AUC_168h_ (h×pg/mL)				
Mean (SD)	6,361 (2,195)	6,344 (2,625)	214,646 (72,342)	231,974 (86,266)
Median (min, max)	6,208 (2,350, 14,959)	5,916 (2,103, 15,094)	207,009 (86,916, 438,122)	221,804 (82,138, 473,168)
Geometric mean	5,992	5828	202,993	216,516
AUC_0–tlast_ (h×pg/mL), n^§^	62	66	63	66
Mean (SD)	6,991 (2,463)	6,897 (2,850)	252,322 (84,387)	271,548 (99,831)
Median (min, max)	6,644 (2,666, 16,934)	6,445 (2,388, 16,254)	236,255 (112,440, 514,555)	238,370 (104,794, 543,705)
Geometric mean	6,574	6,343	239,149	254,411
AUC_240h_ (h×pg/mL), n^§^	62	66	63	66
Mean (SD)	7,086 (2,473)	6,988 (2,854)	252,323 (84,387)	271,549 (99,832)
Median (min, max)	6,790 (2,752, 17,054)	6,538 (2,476, 16,352)	236,255 (112,440, 514,555)	258,370 (104,794, 543,705)
Geometric mean	6,671	6,439	239,150	254,411
AUC_∞_ (h×pg/mL), n^§^	58	63	63	
Mean (SD)	7,213 (2,472)	7,135 (2,800)	259,825 (86,730)	280,545 (102,109)
Median (min, max)	6,866 (2,989, 17,140)	6,632 (2,576, 16,460)	244,748 (117,437, 531,169)	268,795 (109,965, 557,221)
Geometric mean	6,819	6,619	246,478	263,291
C_ss_ (pg/mL)				
Mean (SD)	42.5 (14.7)	41.7 (16.7)	1,410 (473)	1,522 (548)
Median (min, max)	40.6 (17.0, 103)	39.3 (14.9, 98.8)	1,337 (603, 2,878)	1,480 (588, 3,015)
Geometric mean	40.1	38.5	1,335	1,429
T_1/2_ (h), n^§^	58	63		
Mean (SD)	16.1 (4.9)	15.7 (4.6)	27.2 (7.1)	26.8 (6.3)
Median (min, max)	15.4 (7.7, 30.3)	14.5 (7.5, 33.5)	26.7 (15.5, 49.5)	26.5 (14.7, 42.8)
Geometric mean	15.5	15.0	26.4	26.1

*Participants who completed both treatment periods and had PK profiles that allowed for accurate calculation of the PK parameter. ^†^Single application of the currently marketed EVRA patch using Oppanol B100 at beginning of shelf life. ^‡^Single application of the transdermal contraceptive patch using Oppanol N100 at end of shelf life. ^§^R^2^_adj_ < 0.90, or %AUC_∞_ex_ > 20.00%, or parameter not estimated due to missing concentration, or unexpected increase in plasma concentrations at end of PK profile, or could not be estimated. %AUC_∞_ex_ = %AUC extrapolated; AUC_x_ = area under the concentration time curve from time 0 (patch application) to a specified timepoint; C_max_ = maximum observed plasma concentration; C_ss_ = mean steady-state concentration; PK = pharmacokinetics; R^2^_adj_ = adjusted coefficient of determination; SD = standard deviation; T_1/2_ = time to half- life; t_max_ = time to maximum plasma concentration.

**Table 3.** Statistical analysis of PK parameters of ethinyl estradiol and norelgestromin after single, 7-day application of EVRA (PK statistical analysis set*).

	Geometric Least Squares Means			
	Reference patch^†^	Test patch^‡^	Geometric mean ratio (90% CI)	Intra-participant CV (%)
Ethinyl estradiol				
Participants	64^§^	64^§^		
C_max_	47.4	44.5	93.93	19.8
C_ss_ (pg/mL)	40.1	37.6	93.78	13.5
AUC_168h_ (h×pg/mL)	5,999	5,687	94.80	14.7
AUC_0–tlast_ (h×pg/mL)	6,585	6,196	94.09	14.9
AUC_∞_ (h×pg/mL)	6,842	6,485	94.79	12.9
Norelgestromin				
Participants	64^||^	64^||^		
C_max_	1,572	1,615	102.76	13.4
C_ss_ (pg/mL)	1,335	1,405	105.25	11.1
AUC_168h_ (h×pg/mL)	202,923	212,534	104.74	11.4
AUC_0–tlast _(h×pg/mL)	237,241	250,448	105.57	11.3
AUC_∞_ (h×pg/mL)	246,415	259,160	105.17	11.5

*Participants who completed both treatment periods and had PK profiles that allowed for accurate calculation of the PK parameters used in statistical analysis for bioequivalence for both ethinyl estradiol and norelgestromin. ^†^Single application of the currently marketed EVRA patch using Oppanol B100 at beginning of shelf life. ^‡^Single application of the transdermal contraceptive patch using Oppanol N100 at end of shelf life. ^§^n = 62 for AUC_0–tlast_ and n = 55 for AUC_∞_. ^||^n = 62 for AUC_0–tlast_ and n = 63 for AUC_∞_. Note: Log transformed PK parameters were analyzed by mixed-model analysis of variance with treatment, treatment period, treatment sequence, and application site (left or right) as fixed effects and participant as a random effect. Results were back-transformed using anti-logarithm. AUC_x_ = area under the concentration time curve from time 0 (patch application) to a specified timepoint; CI = confidence interval; C_max_ = maximum observed plasma concentration; C_ss_ = mean steady-state concentration; CV = coefficient of variation; PK = pharmacokinetics.

**Table 4.** TEAEs occurring in ≥ 5% of participants in any treatment group (safety set*).

	Reference patch^†^	Test patch^‡^	Overall
Participants	66	68	68
Participants with ≥ 1 TEAE^§^, n (%)	52 (78.8)	56 (82.4)	62 (91.2)
System Organ Class Preferred Term^||^			
Reproductive system and breast disorders	26 (39.4)	23 (33.8)	36 (52.9)
Breast pain	12 (18.2)	13 (19.1)	20 (29.4)
Dysmenorrhea	5 (7.6)	5 (7.4)	8 (11.8)
Breast enlargement	5 (7.6)	1 (1.5)	5 (7.4)
Metrorrhagia	4 (6.1)	2 (2.9)	4 (5.9)
Gastrointestinal disorders	20 (30.3)	24 (35.3)	30 (44.1)
Nausea	8 (12.1)	12 (17.6)	15 (22.1)
Abdominal pain	5 (7.6)	8 (11.8)	11 (16.2)
General disorders and administration-site conditions	17 (25.8)	22 (32.4)	28 (41.2)
Application-site erythema	10 (15.2)	9 (13.2)	14 (20.6)
Fatigue	4 (6.1)	7 (10.3)	11 (16.2)
Application-site pruritus	6 (9.1)	3 (4.4)	7 (10.3)
Nervous system disorders	19 (28.8)	12 (17.6)	23 (33.8)
Headache	16 (24.2)	11 (16.2)	21 (30.9)
Psychiatric disorders	7 (10.6)	13 (19.1)	17 (25.0)
Mood swings	3 (4.5)	4 (5.9)	6 (8.8)
Depressed mood	0	4 (5.9)	4 (5.9)
Musculoskeletal and connective tissue disorders	6 (9.1)	5 (7.4)	8 (11.8)
Back pain	5 (7.6)	3 (4.4)	7 (10.3)

*All randomly assigned participants who received ≥ 1 patch application and had adhesion ≥ 0% at baseline. ^†^Single application of the currently marketed EVRA patch using Oppanol B100 at beginning of shelf life. ^‡^Single application of the transdermal contraceptive patch using Oppanol N100 at end of shelf life. ^§^Participants were counted only once for any given event regardless of the number of times they experienced the event. ^||^TEAEs were coded using Medical Dictionary for Regulatory Activities Version 21.1. TEAE = treatment-emergent adverse event.
